# Atmospheric Pressure Non-Equilibrium Plasma as a Green Tool to Crosslink Gelatin Nanofibers

**DOI:** 10.1038/srep38542

**Published:** 2016-12-07

**Authors:** Anna Liguori, Adriana Bigi, Vittorio Colombo, Maria Letizia Focarete, Matteo Gherardi, Chiara Gualandi, Maria Chiara Oleari, Silvia Panzavolta

**Affiliations:** 1 Advanced Mechanics and Materials, Interdepartmental Center for Industrial Research (AMM-ICIR), Alma Mater Studiorum-University of Bologna, Via Saragozza 8, 40123 Bologna, Italy; 2 Department of Chemistry “G. Ciamician”, via Selmi 2, 40126 University of Bologna, Italy; 3 National Consortium of Materials Science and Technology (INSTM, Bologna RU), University of Bologna, Via Selmi 2, 40126 Bologna, Italy; 4 Department of Industrial Engineering (DIN), Alma Mater Studiorum—Università di Bologna, Via Saragozza 8, 40123 Bologna, Italy; 5 Health Sciences and Technologies – Interdepartmental Center for Industrial Research (HST-ICIR), University of Bologna, Via Tolara di Sopra 50, 40064 Ozzano dell’Emilia, Bologna, Italy

## Abstract

Electrospun gelatin nanofibers attract great interest as a natural biomaterial for cartilage and tendon repair despite their high solubility in aqueous solution, which makes them also difficult to crosslink by means of chemical agents. In this work, we explore the efficiency of non-equilibrium atmospheric pressure plasma in stabilizing gelatin nanofibers. We demonstrate that plasma represents an innovative, easy and environmentally friendly approach to successfully crosslink gelatin electrospun mats directly in the solid state. Plasma treated gelatin mats display increased structural stability and excellent retention of fibrous morphology after immersion in aqueous solution. This method can be successfully applied to induce crosslinking both in pure gelatin and genipin-containing gelatin electrospun nanofibers, the latter requiring an even shorter plasma exposure time. A complete characterization of the crosslinked nanofibres, including mechanical properties, morphological observations, stability in physiological solution and structural modifications, has been carried out in order to get insights on the occurring reactions triggered by plasma.

Gelatin can be obtained from partial hydrolysis of native collagen, which represents the most abundant structural protein of animal tissues^1^. Due to its interesting features, such as biocompatibility, biodegradability, non-immunogenicity and commercial availability at a relatively low cost, gelatin is widely applied in a variety of different fields, including regenerative medicine and tissue engineering^2^,^3^, food engineering, packaging and drug delivery^4^. On the other hand, the main drawbacks of gelatin are its poor mechanical properties and water resistance. In fact, in most applications gelatin crosslinking is required to prevent its solubilization in aqueous solution.

Crosslinking of gelatin can be performed using either physical methods, such as dehydrothermal treatment, ultraviolet and gamma irradiation, or chemical agents, as diisocyanates, glutaraldheyde, carbodiimides, acyl azide, genipin and polyepoxy compounds. However, the most common crosslinking agents often give rise to cytotoxicity problems, and are therefore not suitable for biomedical applications^5^,^6^,^7^,^8^,^9^,^10^. Many efforts have been devoted to develop new methods to crosslink gelatin without using chemical compounds^11^,^12^. Non-equilibrium plasma, being an environmentally friendly technology, can represent a suitable solution for the achievement of the proposed aim. Some studies have been recently performed to evaluate the possibility of employing a single-step plasma process to induce crosslinking of water-soluble polymers in the liquid phase, exploiting the ability of non-equilibrium plasma treatment to induce the formation of radicals, which may recombine producing unsaturation and polymer crosslinking^13^,^14^,^15^. Molinas *et al*.^16^ reported about the possibility to use an atmospheric dielectric barrier discharge (DBD) plasma to induce *in-situ* liquid phase gelation of an aqueous solution of chitosan in order to obtain a crosslinked hydrogel. Moreover, efficient crosslinking of starch slurries by glow discharge plasma has been reported^17^. Interestingly, the efficiency of plasma to induce crosslinking of polymers in liquid phase was also evaluated on a gelatin solution. In particular, Prasertsung *et al*.^18^ pointed out that the generation of pulsed electrical discharges in gelatin solutions enables: (i) to increase the solution viscosity; (ii) to enhance the gel strength; (iii) to produce films characterized by a higher crosslinking degree with respect to those obtained by untreated gelatin solutions.

To the best of our knowledge, only few studies report the use of low pressure plasma to confer water resistance and improve the mechanical properties of gelatin directly in the solid state^19^,^20^. These studies, carried out on electrospun gelatin fibers, put into evidence that low pressure plasma treatments were not successful in stabilizing gelatin against water solubilization. In particular, Ratanavaraporn *et al*.^19^ investigated the effects of several physical and chemical methods for the crosslinking of electrospun gelatin (type A and B) mats and they found that plasma treatment, carried out by means of a pulsed inductively coupled plasma operating in Ar at a pressure of 5 bar, induced a crosslinking degree of 45% on type A gelatin, and an even lower degree on type B gelatin. Furthermore, SEM analysis performed on plasma treated mats highlighted melted fibers at the surface, underlining that the plasma treatment thermally affected the surface of the mats. The Authors concluded that plasma treatments, performed in the employed conditions, involved only the surface fibers resulting in a limited crosslinking degree.

Sisson *et al*.^20^ reported the use of different methods to crosslink electrospun gelatin scaffolds. Among the others, the exposure to a plasma cleaner operating in oxygen at low pressure was also tested. The Authors pointed out that, despite no visible alteration of fibers morphology was detected after the plasma exposure, the crosslinking degree turned out to be rather low, since the scaffold degraded after only a few hours of immersion in buffer solution at 37 °C.

The present work investigates the efficiency of a method based on the use of the atmospheric pressure non-equilibrium plasma generated by a DBD operated in open air to crosslink electrospun gelatin mats, paving the way to the possibility of effectively using this approach for crosslinking without requiring chemical agents.

In addition, we explored the influence of plasma treatment on electrospun gelatin fibers containing genipin, a natural crosslinking agent previously employed by some of the Authors to successfully crosslink gelatin electrospun mats in combination with properly optimized thermal and solvent treatments^10^. The role of plasma treatment time on the structural stability of mats and their retention of morphological structure upon contact with an aqueous solution was investigated. Furthermore, different extent of the crosslinking reactions triggered by plasma was achieved through immersion in two different aqueous solutions, namely double distilled water (DDW) and phosphate buffer pH 7.4 (PB). The chemical investigation of the plasma treated mats was carried out by means of ATR-FTIR spectroscopy and quantitative evaluation of the crosslinking extent, in order to get preliminary insight into the reaction mechanisms. SEM images were collected to investigate the effect of the plasma exposure on the fibers morphology before and after the immersion of the mats in aqueous solutions. To get further proofs regarding the occurred crosslinking, mats were also subjected to mechanical analysis before and after plasma exposure.

## Results and Discussion

Since gelatin is highly soluble in aqueous solution, several applications require its crosslinking in order to confer water stability and prevent solubilization.

In the present work, electrospun pure gelatin (G) and genipin-containing gelatin mats (GG) were crosslinked directly in the solid state, by means of an innovative, environmentally friendly and easy to use method, consisting in exposing mats to an atmospheric pressure non-equilibrium plasma treatment^21^, and then in rinsing them in aqueous solution to stabilize the crosslinking reactions triggered by plasma onto the fibers surface. The labels of the samples subjected to the different treatment are reported in Table 1.

**Figure 1:**
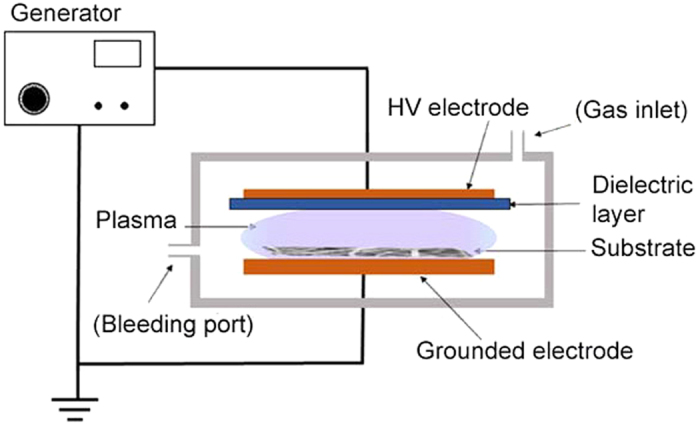
Schematic of the DBD plasma source employed.

The DBD plasma source employed in this work is reported in Fig. 1. Due to the porous structure of the electrospun mats, the plasma generated in the inter-electrode volume was able to induce crosslinking reactions also onto the surface of the fibres placed across the thickness of the mat^22^.

### Mat characterization

**Figure 2:**
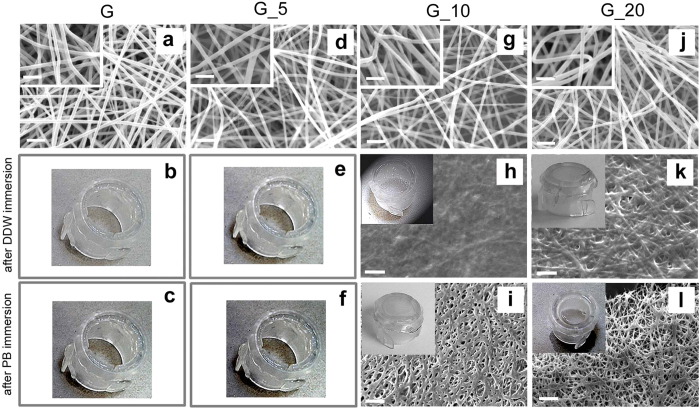
SEM images and pictures of gelatin as-spun mats, gelatin mats after plasma treatment and after immersion either in DDW or PB: (**a**) as-spun G; (**b**) G_W; (**c**) G_B; (**d**) G_5; (**e**) G_5 W; (**f**) G_5B; (**g**) G_10; (**h**) G_10 W; (**i**) G_10B; (**j**) G_20; (**k**) G_20 W; (**l**) G_20B. Scale bars: (**a**,**d**,**g**,**j**) = 2 μm; (**h**,**i**,**k**,**l**) = 5 μm. Inset: magnification of mats G, G_5, G_10 and G_20, respectively. Scale bar = 1 μm.

SEM images of as-spun G mats, shown in Fig. 2a, display defect free and regular fibers. Figure 2(d,g,j) reports SEM images of G mats exposed to plasma for 5, 10 and 20 minutes, respectively.

The insets show the fibers at a higher magnification, highlighting that plasma treatment did not provoke any noticeable - morphology alteration. In agreement, the measured diameter of the fibers, 320 ± 40 μm, did not vary after plasma exposure independently of the treatment time. This result highlights that the employed non-equilibrium atmospheric pressure plasma does not alter the morphology of gelatin fibers differently from the low pressure plasma process, adopted by Ratanavaraporn *et al*., which thermally affected the surface fibers of the mat, leading to their melting. In order to evaluate the capability of plasma treated mats in preserving fiber morphology upon liquid contact, G samples were fixed to plastic rings after plasma treatment and immediately rinsed in aqueous solution (DDW or PB). As shown in Fig. 2b and c, the as-spun G mats dissolved immediately upon DDW or PB contact, while plasma treated G mats better resisted to solubilization when the proper treatment time was selected.

In particular, for constant plasma operating parameters, the longer the treatment time the higher the stability in solution and the preservation of fiber morphology (compare Fig. 2e,h,k,f,i,l). This finding is in agreement with previous results obtained on plasma treated electrospun polysaccharides^21^ and demonstrates that there is a correlation between plasma treatment time and the crosslinking degree.

In details, G mats plasma treated for 5 minutes (G_5) do not exhibit any relevant increased stability, being immediately dissolved when in contact with aqueous solution (see Fig. 2e and f). Conversely, a treatment time of 10 minutes improves G mats stability and avoids their solubilization (see Fig. 2h and i). However, the fibrous structure is lost and the porous mat is turned into a continuous film when immersed in DDW, whereas the porous structure is partially maintained after soaking in PB. The increase of treatment time up to 20 minutes provides a further improvement in mat stability and retention of fiber morphology after immersion in both DDW and PB (compare Fig. 2k and l), with a better preservation of the nanofibrous structure in PB than in DDW. The increased stability of the mat when soaked in PB is consistent with the higher pH value of the buffer solution with respect to the slightly acidic DDW and with the reported reduced swelling of gelatin gels in PB with respect to water^23^, which has been ascribed to the higher ionic strength and consequently to the higher osmotic pressure of PB.

**Figure 3:**
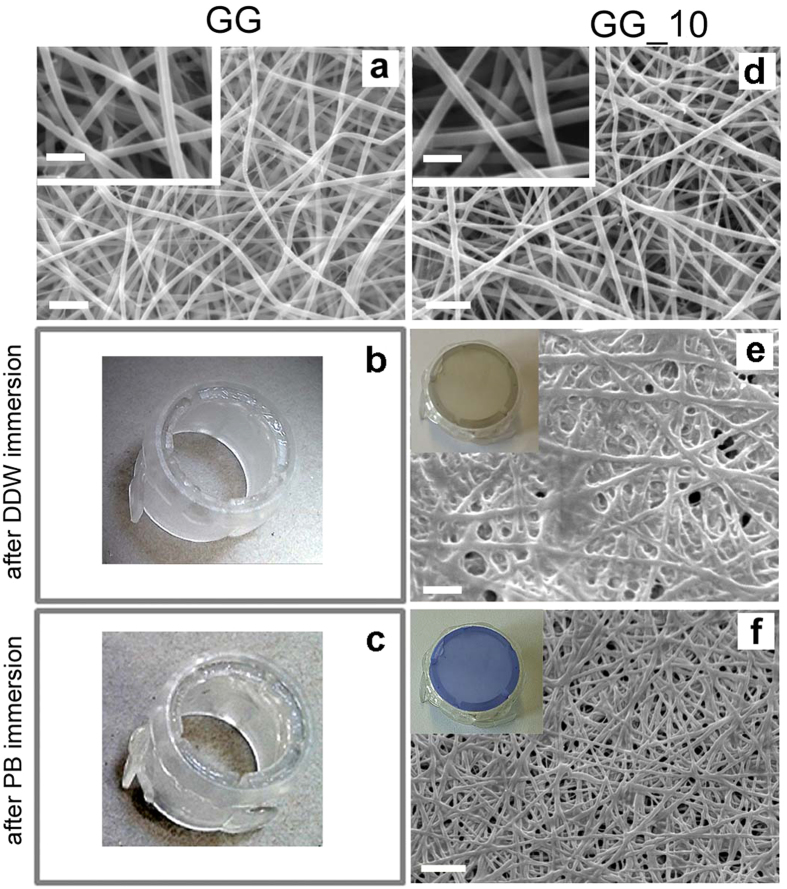
SEM images and pictures of genipin-containing gelatin as-spun mats, genipin-containing gelatin mats after plasma treatment and after immersion either in DDW or PB: (**a**) as-spun GG; (**b**) GG_W; (**c**) GG_B; (**d**) GG_10; (**e**) GG_10 W; (**f**) GG_10B. Scale bars: (**a**,**d**,**e**) = 2 μm; (**f**) = 5 μm. Inset: higher magnification of mats GG and GG_10. Scale bar = 1 μm.

In order to further improve the stabilization of as electrospun mats, the natural crosslinker genipin was introduced in gelatin fibers during electrospinning, as reported elsewhere^24^. SEM images of electrospun GG mats before and after 10 minutes of plasma treatment are reported in Fig. 3a and d, respectively.

As for G samples, plasma treatment does not influence the morphology of the fibers, which exhibit a mean diameter of 355 ± 55 μm, not significantly different from that of GG samples. In absence of plasma treatment, the presence of genipin does not prevent the solubilization of GG mats when immersed either in DDW or PB (Fig. 3b and c), indicating that the amount of genipin and the contact time between gelatin and genipin during electrospinning are not sufficient to stabilize the mats.

On the contrary, GG samples submitted to plasma treatment for 10 minutes preserve their fibrous morphology after immersion in water and, even more evidently, in PB (compare Fig. 3e and f).

The shorter plasma treatment time required to stabilize GG mats with respect to G mats indicates that genipin has a relevant role in preserving the fibrous morphology and suggests that plasma treatment activates gelatin crosslinking mediated by genipin, which is further promoted by PB, as shown by the brownish and blue color^25^ of GG_10W and GG_10B (insets Fig. 3e and f), respectively.

Considering the above described results, further characterizations were carried out only on selected samples, namely on electrospun G and GG mats plasma treated for 20 and 10 minutes, respectively.

The extent of crosslinking of G and GG samples was determined for as-spun mats and for plasma treated ones by using a method based on the evaluation of the moles of unreacted ε-amino groups per gram of gelatin, according to Equation 1, as reported in the Experimental Part. The number of ε-amino groups of G samples did not change before and after plasma treatment, indicating that no oxidation of amino groups was induced by plasma. On the other hand, a significant decrease of ε-amino groups was observed after immersion in PB (G_20B), with an associated increase of crosslinking degree from 0% to 61%, in agreement with the observed conservation of the fibrous structure exhibited by G_20B.

The reduction of the number of ε-amino groups for G_20B suggests that PB promoted the formation of covalent bonds involving reactive groups originated by plasma treatment and ε-amino groups of the gelatin. As-spun GG and G samples display the same number of ε-amino groups, indicating that the simple addition of genipin within the fibers during electrospinning does not induce the formation of appreciable crosslinking bonds. This finding is not surprising since for obtaining the results presented in this work we used a specifically designed coaxial needle (described in the Materials and methods section) which allows the simultaneous electrospinning of different solutions, at the desired values of feeding rate, concentration and mixing time: due to the relatively short time of contact between gelatin and genipin during the electrospinning process, the crosslinking reactions between gelatin and genipin did not occur. Suitable contact time between gelatin and the crosslinking agent is needed to yield crosslinking reaction, as it occurs by electrospinning a genipin-gelatin solution^10^. However, differently from G_20, GG_10 mats exhibited a significant decrease of ε-amino groups, consistent with a crosslinking degree of 36%. This result is quite amazing and indicates that plasma is effective in initiating, in the solid state, crosslinking reactions that involve free ε-amino groups, similarly to the conventional crosslinking reactions induced in the liquid phase by genipin. Moreover, immersion in PB had a great influence on the stabilization and enhancement of crosslinking degree even for GG samples. In fact, immersion in PB provoked an increase of the extent of crosslinking of GG_10 mats from 36% up to 100% (see Table 2). This outcome is in tune with the reported positive contribution of PB to the crosslinking reaction of genipin^26^, which displays its maximum crosslinking activity at pH 7.4^27^.

**Figure 4:**
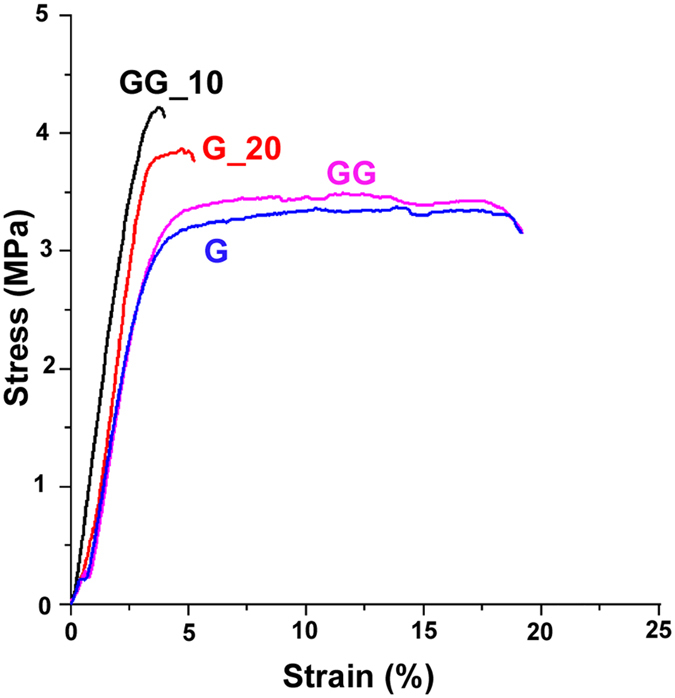
Typical stress-strain curves recorded from as-spun G and GG samples and from G_20 and GG_10 samples.

The mechanical behavior of as-spun G and GG samples was evaluated by tensile stress-strain measurements and compared with those of G_20 and GG_10 samples in order to investigate the effect of the plasma treatment on the mechanical properties of the samples (Fig. 4).

Typical stress-strain curves are reported in Fig. 4, whereas the mean values of Young’s Modulus, stress at break (σ_b_) and strain at break (ε_b_) obtained for the considered samples are reported in Table 3.

No significant differences were appreciated between the mechanical behaviors of as-spun G and GG mats, suggesting that the presence of genipin did not induce any remarkable effect on the mechanical parameters. Conversely, the plasma exposure leads to a significant reduction of the deformation at break (ε_b_), for both the considered types of samples, whereas the values of elastic modulus (E) and stress at break (ε_b_) are higher, even though not significantly different, than those of as-spun mats.

Despite the different number of free ε-amino groups, the similar values of the mechanical parameters obtained for G_20 and GG_10 mats suggest that plasma treatment induces the formation of reactive groups able to create physical and/or chemical crosslinks that contribute to gelatin stabilization also without involving ε-amino groups.

### Mechanism

Elucidating the mechanisms of plasma-induced crosslinking of gelatin nanofibers is not a simple issue, especially considering that only few papers report on the application of plasma to a gelatin based material^19^,^20^. Moreover, these studies were carried out by using low pressure plasmas, which did not succeed in obtaining highly stabilized gelatin samples.

In order to investigate if the increase of the temperature of the mat during the plasma exposure could have a crosslinking effect, this parameter was monitored, as described in the Methods. Some of the Authors previously demonstrated that heating at 55 °C for 24 h can promote the crosslinking of GG mats, while no crosslinking can be induced by this method on G mats^24^. Moreover, it was also reported that the heating of gelatin nanofibres at 140 °C for 48 h, followed by plasma treatment, is not able to induce any satisfactory result in terms of crosslinking^19^. Since the achieved results highlighted that after 20 minutes of plasma exposure, the temperature passed from 23 ± 1 °C (room temperature) to 31 ± 2 °C, underlining a negligible heating of the mats during the process, the crosslinking of the G and GG mats cannot be attributed to any thermal effect. Subsequently, the synergic role of the active components of the air plasma, such as electrons, ions, radicals, UV radiation, turns out to be decisive. The analysis of the active species present in a non-equilibrium atmospheric pressure plasma, generated by a DBD operated in environmental air have been widely performed in scientific literature^28^,^29^,^30^, by means of specific techniques to identify the plasma content, such as optical emission spectroscopy, mass spectroscopy, gas detection and UV absorption spectroscopy. The reported results underline that air plasmas are excellent sources of reactive oxygen species and reactive nitrogen species, such as atomic oxygen, ozone, hydroxyl and NOx, in particular NO, NO_2_, etc^28^,^29^,^30^.

Several of these components might play a role in plasma-induced crosslinking. In particular, previous works reported that UV could lead to gelatin intermolecular crosslinking and, possibly, to the formation of radicals on the aromatic residues of collagen and gelatin amino acids, such as tyrosin and phenylalanine^31^,^32^. Besides UV radiation, the electrons of plasma also interact with the substrate, provoking a nearly instantaneous formation of radicals into the polymeric chains and starting the crosslinking. Apart from this reaction, chain scission can occur simultaneously leading to radiolytic degradation processes in the polymeric matrix^33^. However, since no degradation of gelatin was observed after plasma exposure, the latter effect can be excluded and electrons are to be considered only responsible of contributing to the formation of radicals in the polymeric chains. The polymeric radicals generated by the plasma components can interact with the OH species, leading to the formation of hydroxyl compounds onto the polymeric fibers of the mats, which, in turn, lead to the increase of the number of crosslinking junctions through hydrogen bonding, as previously found by Fijitsu *et al*. in gelatin gels^34^.

**Figure 5:**
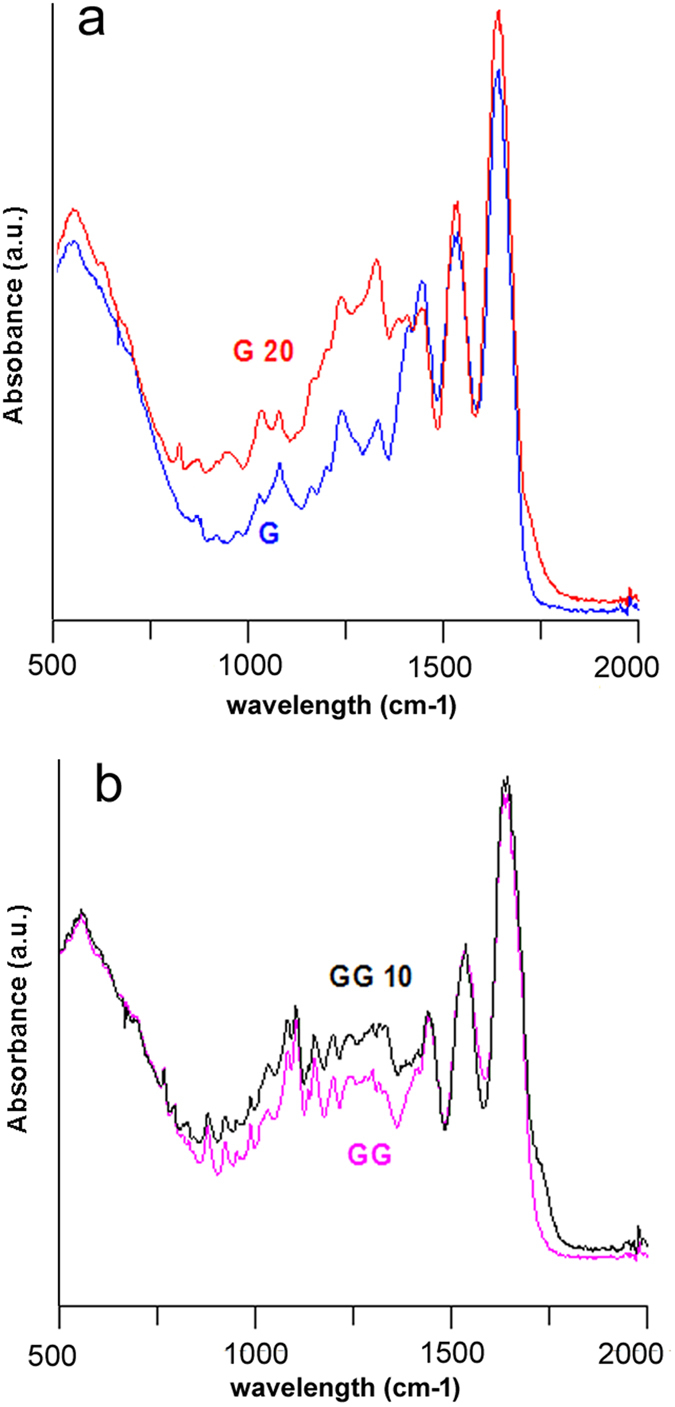
FT-IR absorption spectra collected on samples (**a**) G, G_20; (**b**) GG, GG_10.

In order to evaluate if plasma treatment induces some structural changes on gelatin-based mats, we performed an ATR-FTIR analysis of samples G, G_20, GG and GG_10. Infrared spectrum of as-spun gelatin presents several absorption bands corresponding to Amide I, II and III^10^. Figure 5 reports the relevant part of the spectra of the samples before and after plasma treatment. Comparing the spectra of G and G_20 (Fig. 5a), plasma treatment is evidently responsible for an increase of the relative intensity of the bands in the 1300–1180 cm^−1^ range, corresponding to Amide III absorption^35^. A similar effect of plasma treatment can be appreciated also on comparing the absorption spectra of GG and GG_10 (Fig. 5b), although in this case the difference between the relative intensity of the Amide III bands is less evident, most likely due to the genipin contribution to the absorption spectra^36^. Since the 1300–1180 cm^−1^ range of the infrared spectrum of collagenous material includes C-O stretching and N-H bending vibrations of Amide III, the observed increase of intensity observed on plasma treated mats supports the hypothesis of formation of H bonds among the reactive species created by plasma treatment.

The stability in solution of the mats over time was tested by immersing G_20B and GG_10B samples in PB at 37 °C for different periods of time. In a previous study, Sisson *et al*.^20^ used a low pressure plasma operating in oxygen, but did not achieve a lasting mats stability, since the scaffold completely dissolved after only 12 h of immersion in DMEM at 37 °C. In our case, after 1 day of soaking, the amount of released gelatin is about 5% (w/w) for GG_10B: the release increases by increasing the soaking time and reaches a value of about 30% (w/w) after 7 days of immersion. On the other hand, G_20B sample releases a higher amount of gelatin after 1 day (10%) and it dissolves completely after 7 days of soaking. These results fit very well with the values of the crosslinking degree in Table 2: GG_10B displays the higher crosslinking extent and, consequently, dissolves slower when immersed in aqueous solution.

### Aging

Plasma treated polymeric materials and solutions are well-known to undergo a recovery of their chemical characteristics over storage time, probably due to post-plasma oxidation processes caused by the radicals still present on the material surface or to the recovery of the polymer chains from the surface into the bulk of the materials^37^,^38^,^39^. The ageing effect on plasma-treated electrospun mats was tested by dipping the samples in PB after 15 days from their exposure to plasma. After immersion, the samples were dried at room temperature and their morphology was observed at SEM.

**Figure 6:**
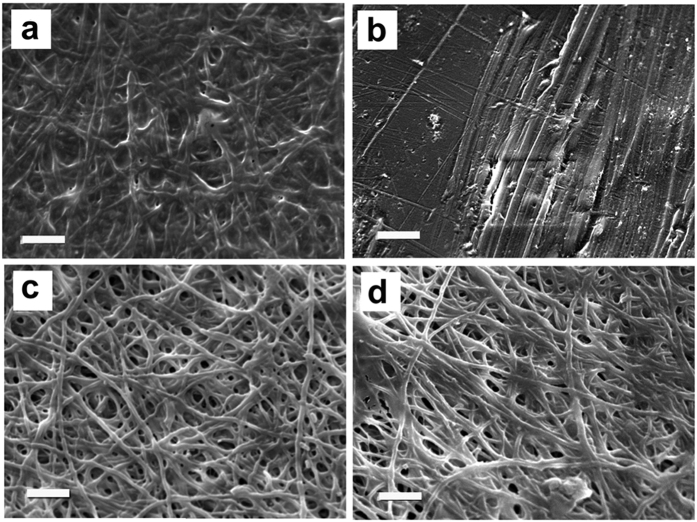
Effect of ageing on plasma treated mats: (**a**) GG_10 and (**b**) G_20 both immersed in PB 15 days after plasma exposure; (**c**) GG_10B and (**d**) G_20B immersed in PB 15 days after their preparation. Scale bars: (**a–d**) = 5 μm.

The images of mats surfaces (Fig. 6a and b, respectively) show that soaking GG_10 and G_20 in PB 15 days after plasma treatment caused partial solubilization and loss of the nanofibrous morphology. On the other hand, the nanofibrous structure of samples GG_10B and G_20B was maintained (see Fig. 6c and d). The better retention of fibrous morphology with time, shown by GG_10B and G_20B with respect to GG_10 and G_20, confirms that the immersion in PB immediately after plasma treatment is mandatory to prevent the loss of stability over time. The chemical/physical interactions between the polymeric chains of the plasma-treated samples and the solution, which contribute to the gelatin structural stability, are probably due to the radicals and active sites generated by plasma in the polymeric chains of the mats. The plasma-generated radicals and active sites can recombine with aging, which justifies the poor retention of fiber morphology observed for GG_10 and G_20. At variance, PB immersion stabilizes these interactions and prevents the recombination of the plasma-generated radicals and active sites over time.

## Conclusions

The data show that non-equilibrium atmospheric pressure plasma treatment can successfully induce the crosslinking of gelatin electrospun mats, as well as of genipin-containing gelatin mats directly in the solid state. It has been demonstrated that plasma treatment triggers crosslinking reactions, as highlighted by the observed decrease of the deformation at break, as well as by the structural and morphological stability of electrospun mats soaked in aqueous solution after plasma exposure. Plasma treatment provoked an increase of the crosslinked ε-amino groups. However, stabilization of the mats must, at least partly, be ascribed also to chemical/physical mechanisms not involving the gelatin free amino groups. Interestingly, immersion in PB, performed immediately after plasma exposure, was demonstrated to be necessary to appreciate plasma crosslinking of pure gelatin mats, and further stabilize the genipin-gelatin mats, providing highly crosslinked materials that retain their stability over time.

## Methods

### Materials

Type A gelatin (300 Bloom) from porcine skin was supplied by Sigma-Aldrich, genipin was provided by Wako Chemicals USA and was used without further purification. Acetic acid (AcAc) was supplied by Sigma-Aldrich.

### Preparation of Genipin-Containing Gelatin Electrospun Mats

The electrospinning apparatus, made in house, is composed of a high voltage power supply (Spellman SL 50P 10/CE/230), two syringe pumps (KD Scientific 200 series), a glass syringe, a stainless steel coaxial needle connected to the power supply electrode and a grounded aluminum collector (10 cm × 10 cm). The coaxial needle used in the present work enables to introduce a given amount of genipin in the fibers avoiding gelatin gelling; in brief, an inner needle (O.D. = 0.9 mm, I.D. = 0.6 mm) is positioned concentrically to the outer needle (O.D. = 1.5 mm, I.D. = 1.2 mm). The tip of the outer needle protruded 10 cm below that of the inner needle. In addition, the tip of the inner needle wall presents twenty little holes of 0.5 mm in diameter organized in four lines distributed along the needle circumference. Each line contains five little holes 2 mm apart from each other. The solutions were individually dispensed at a controlled flow rate by using the syringe pumps through a Teflon tube to the outer and inner needles. The flow rate of the shell solution was set at 0.18 ml/h while that of the core solution was 0.09 ml/h. The coaxial needle was placed vertically to the collecting plate at a distance of 15 cm, with respect to the outer needle, and the applied voltage was set at 21 kV. The shell solution was prepared by dissolving gelatin in AcAc:H_2_O (60:40 V/V) at a concentration of 30% (w/V). To fabricate gelatin mats containing 18% (w/w) of genipin (labeled GG), the core solution was prepared by dissolving genipin at a concentration of 12% (w/V) in AcAc, as reported elsewhere^24^. A reference mat not containing genipin (labeled G) was prepared by using a stainless steel blunt-ended needle (inner diameter 0.84 mm) connected to the power supply electrode, as reported elsewhere^24^. Electrospun mats were kept under vacuum over P_2_O_5_ at room temperature (RT) overnight in order to remove residual solvents and removed from the desiccator before characterizations and plasma treatments.

### Atmospheric Pressure Non-equilibrium Plasma Treatment

The Dielectric Barrier Discharge (DBD) plasma source employed in this work (reported in Fig. 1) consists of two aluminum parallel-plate electrodes; the upper electrode, having a surface of 15 × 10 cm^2^ and a thickness of 0.13 mm, is connected to the high voltage (HV) generator, while the lower electrode, with a surface of 15 × 9 cm^2^ and a thickness of 0.13 mm, is grounded. As dielectric, a POM-C plate, having a surface of 19 × 15 cm^2^ and a thickness of 2.4 mm, covers the upper electrode. A gap of 1 mm between the grounded electrode and the POM-C plate was used for all plasma treatments. The plasma source is enclosed in a volume having a size of (21 × 17 × 3) cm^3^ (L × W × H). For the proposed process, plasma generation was performed in environmental air with no introduction of additional gases inside the plasma chamber and keeping the gas inlet and the bleeding port, placed on its top and its side, respectively, open during the process. The air temperature was 23 ± 1 °C, while its relative humidity was 45%. Since the gas inlet and the bleeding port could be used to introduce specific gases and to bleed out air during the initial flushing, the chamber is also suitable for other processes aimed at conferring specific chemical properties to the substrate subjected to the plasma treatment.

The DBD plasma source was driven by a micropulsed generator, producing high voltage bursts (duration 7 ms) with a burst repetition frequency (BRF) of 125 Hz; during the bursts a 20 kHz sinusoidal waveform with 12 kV peak voltage is produced. In order to perform the plasma treatment on electrospun samples, mats were placed on the grounded electrode and directly subjected to the plasma discharge.

During the process, the temperature of the mats subjected to plasma treatment was monitored by means of a fiber optic temperature sensor (OPSENS OTP-M) with a calibration range of 20 °C–60 °C, a resolution of 0.01 °C, an accuracy of 0.15 °C, and a response time of less than 1 s. The measurements were performed by placing the fiber optic sensor head in contact with the mat surface, while a second fiber optic sensor was employed to monitor the room temperature.

G samples (5 × 5 cm^2^, width 45 μm) were treated for different times (5, 10 and 20 minutes) and were labeled G_5, G_10 and G_20, accordingly, while GG samples (5 × 5 cm^2^, width 55 μm) were treated for 10 minutes (GG_10). After plasma discharge, a part of the treated samples was transferred into a desiccator while parts were fixed to plastic rings (CellCrown™, Scaffdex, diameter = 12 mm) and rinsed either in 10 ml of Double Distilled Water or of Phosphate Buffer (PB, pH 7.4 and Ionic Strength = 0.26 M) for 20 seconds, in order to evaluate the effect of pH and Ionic Strength (I) on the morphological and structural stability of electrospun mats.

Depending on the soaking medium, the samples were labeled either W (in the case of Double Distilled Water) or B (in the case of PB). After immersion, mats were air-dried at RT and transferred to a desiccator prior to further characterizations. Samples differently treated were labeled as reported in Table 1.

### Characterization Techniques

Morphological observations were carried out by using a Philips XL-20 SEM at an accelerating voltage of 15 kV, on samples sputter-coated with gold. The distribution of fiber diameters was determined through the measurement of about 200 fibers by means of an image acquisition and analysis software (EDAX Genesis) and the results were given as the average diameter ± standard deviation.

ATR-FTIR analysis of the electrospun mats was carried out using an Agilent Cary 660 FTIR spectrometer equipped with an ATR sampling device, using a diamond crystal as internal reflection element. Infrared spectra were acquired at room temperature in absorbance mode, from 3900 to 400 cm^−1^ with a resolution of 2 cm^−1^; a total of 32 scans were recorded for each spectrum.

The extent of crosslinking of gelatin mats was determined by a UV assay of uncrosslinked ε-amino groups before and after crosslinking treatment^10^. Electrospun samples of about 5 mg were incubated with 1 ml of a 4% (m/V) NaHCO_3_ solution and 1 ml of TNBS solution at 0.5% (m/V) for 4 hours at 40 °C. 3 ml of HCl 6 M were then added and the solution was maintained at 110 °C for 24 hours. The absorbance of the diluted solution was measured at 346 nm in a Kontron Uvikon 931 spectrophotometer against a blank. Equation 1 relates the solution absorbance and moles of ε-amino groups per gram of gelatin:


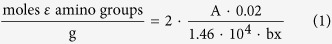


where A is the measured absorbance, 0.02 l is the volume of the analyzed solution, 1.46 × 10^4^ (l × mol^−1^ × cm^−1^) is the molar absorptivity of TNP-lys, b is the cell path length in cm, and x is the mat weight in g. The crosslinking degree is the ratio between the moles of free ε-amino groups in crosslinked gelatin and the moles of ε-amino groups in uncrosslinked gelatin.

The amount of gelatin released by mats kept in PB at 37 °C for different periods of time was measured by using a spectrophotometric method. About 1 mg of electrospun sample was immersed in 1 ml of PB at 37 °C. At defined time intervals the PB was removed and substituted with a fresh one. 200 μl of the extracted solution were incubated at 40 °C for 30 min with 3 ml of PB and 2 ml of a solution containing 4% w/v of aqueous CuSO_4_ and bicinconinic acid in a ratio of 1:50 v/v. The absorbance of the solution was measured at 562 nm in a Kontron Uvikon 931 spectrophotometer against a blank and the gelatin concentration was calculated by using a pre-determined calibration curve^40^. The amount of released gelatin was measured after 24, 48 and 168 hours of immersion in PB.

For the evaluation of mechanical properties, strip-shaped samples (5 × 20 mm, thickness around 45 μm and 55 μm for G and GG samples, respectively) were used. Stress–strain curves of as-spun mats and of mats 24 h after the plasma were recorded using an Instron Testing Machine 4465, with a cross-head speed of 1 mm min^−1^, and the Series IX software package.

Samples were tested in a dry state: the Young’s modulus (E), the strain at break (ε_b_) and the stress at break (σ_b_) of the strips were measured.

## Additional Information

**How to cite this article**: Liguori, A. *et al*. Atmospheric Pressure Non-Equilibrium Plasma as a Green Tool to Crosslink Gelatin Nanofibers. *Sci. Rep.* **6**, 38542; doi: 10.1038/srep38542 (2016).

**Publisher’s note:** Springer Nature remains neutral with regard to jurisdictional claims in published maps and institutional affiliations.

## Figures and Tables

**Table 1:** Labels of the samples before and after plasma treatment and immersion in DDW (W) or PB (B).

As-spun Samples	After Plasma treatment	After Soaking in PBS	After Soaking in DDW
G	—	G_B	G_W
	G_5	G_5B	G_5 W
	G_10	G_10B	G_10 W
	G_20	G_20B	G_20 W
GG	—	GG_B	GG_W
	GG_10	GG_10B	GG_10 W

The numbers indicate the plasma treatment time (min).

**Table 2:** Number of free ε-amino groups (mol/g gelatin) and extent of crosslinking calculated as reported in EQ1.

Sample	N° of free ε-NH_2_ groups	Crosslinking degree (%)
G	2.2*10^−4^ ± 0.1*10^−4^	0
G_20	2.2*10^−4^ ± 0.1*10^−4^	0
G_20B	8.6*10^−5^ ± 0.2*10^−5^	61 ± 1
GG	2.2*10^−4^ ± 0.2*10^-4^	0
GG_10	1.4*10^−4^ ± 0.1*10^−4^	36 ± 1
GG_10B	n.d.	100

Each value is the mean of three determinations and it is reported with its standard deviation.

**Table 3:** Young’s modulus, E, stress at break, σ_b_, and strain at break, ε_b_, of sample G and GG before and after plasma treatment.

Sample	E (MPa)	σ_b_ (MPa)	ε_b_ (%)
G	120 ± 30	2.5 ± 0.4	20 ± 3
G_20	140 ± 10	3.1 ± 0.3	6 ± 1
GG	120 ± 20	3.1 ± 0.4	18 ± 4
GG_10	160 ± 20	3.8 ± 0.6	6 ± 2

Each value is the mean of seven determinations and is reported with its standard deviations.
